# Latent class analysis of conventional ultrasound features: a novel approach to predicting non-response to neoadjuvant chemotherapy in breast cancer

**DOI:** 10.3389/fonc.2026.1813724

**Published:** 2026-05-07

**Authors:** Mei-Lian Zhang, Yi Tang, Cheng-Hua Kong, Li Sun, Xing-Qing Li, Yu Zhou, Zhi-Kui Chen

**Affiliations:** 1 Department of Ultrasound, Fujian Medical University Union Hospital, Fuzhou, China; 2 Department of Clinical Pharmacy and Pharmacy Administration, School of Pharmacy, Fujian Medical University, Fuzhou, China

**Keywords:** breast cancer, individualized treatment, neoadjuvant chemotherapy, pathological response, ultrasound image features

## Abstract

**Objective:**

To determine if holistic patterns derived from conventional ultrasound features are associated with pathological response to neoadjuvant chemotherapy (NAC) in breast cancer, providing a potential basis for non-invasive treatment monitoring.

**Methods:**

This study included 509 female breast cancer patients with unilateral primary lesions who underwent NAC followed by surgery. We employed three clustering methodologies to integrate conventional ultrasound features into holistic patterns: the model-based latent class analysis (LCA) and the distance-based K-modes and hierarchical clustering. The association between the resulting patterns and pathological non-response was assessed using Firth logistic regression, with internal validation performed through 10-fold cross-validation and 10, 000 bootstrap resampling.

**Results:**

When comparing the three approaches, LCA was identified as the superior classification framework, providing the best balance of statistical model fit, class separation, and meaningful association with clinical outcomes. The optimal LCA model identified two distinct sonographic patterns, referred to as Pattern A and Pattern B. Pattern B was significantly associated with a 2.51-fold higher (95% CI: 1.05-5.99) likelihood of pathological non-response compared to Pattern A. In subgroup analyses, among defined high-risk groups, Pattern B was linked to an increased risk of pathological non-response ranging from 12.84% (95% CI: 5.74%–23.59%) to 21.34% (95% CI: 13.63%–28.32%), whereas in non-high-risk groups the corresponding increase ranged from 1.12% (95% CI: 0.79%–1.51%) to 3.45% (95% CI: 2.60%–4.47%).

**Conclusion:**

Patterns of conventional ultrasound features derived through LCA may offer a preliminary indication for identifying breast cancer patients less likely to benefit from NAC, potentially informing more individualized treatment planning following external validation.

## Introduction

Breast cancer remains a major global health burden, with 2.31 million new cases reported worldwide in 2022 (11.6% of female cancers) and approximately 670, 000 deaths (15.4% of female cancer mortality) (1). Neoadjuvant chemotherapy (NAC) is widely used to downstage tumors and enable surgical management (2, 3). Despite its clinical value, NAC is not uniformly effective and may lead to significant adverse drug reactions, including myelosuppression and organ toxicity (4–6). Consequently, clinical care requires strategies that can identify patients unlikely to benefit from NAC in a timely manner, so that treatment can be reconsidered before cumulative harm from ineffective therapy occurs.

Non-invasive monitoring is therefore essential. Ultrasound is a practical modality for longitudinal assessment because it is radiation-free, available in real time, and relatively low cost. Recent advances have explored ultrasound -based approaches for pathological complete response (pCR) prediction; for example, multimodal AI models integrating quantitative imaging features and clinical variables have demonstrated high predictive accuracy in triple-negative breast cancer (TNBC) populations (7), and deep learning-based models have shown promise in predicting axillary pCR with improved clinical decision-making for surgery (8). However, these AI-driven approaches often face implementation challenges related to pixel-level feature extraction and the standardization of image processing pipelines. In contrast, conventional ultrasound remains attractive because it provides multidimensional descriptors of treatment-related change, including size (9), internal echos (10, 11), blood flow patterns (12, 13), and boundary regularization (14, 15). In routine practice, clinicians typically integrate these findings as patterns rather than interpreting each parameter independently.

At the same time, breast imaging is evolving. Emerging modalities such as optical coherence tomography, Raman spectroscopy, photoacoustic imaging, and hyperspectral approaches have shown improved diagnostic performance in reported studies, but broader clinical adoption remains limited by practical barriers including validation, standardization, and workflow integration (16). For hyperspectral imaging-based computer-aided detection, recent evidence summarized in a systematic meta-analysis indicates promising diagnostic accuracy while also highlighting ongoing heterogeneity across study designs and settings (17). Within this context, studies that leverage routinely obtained imaging data may better align with near-term clinical translation.

Most existing prediction studies have focused on pCR as the endpoint. However, conventional ultrasound has shown limited performance for pCR prediction (18, 19), partly because pCR rates can be relatively high and the imaging differences between patients who achieve pCR and those who do not may be subtle. An alternative, clinically actionable task is to identify pathological non-responders—patients who exhibit substantial residual invasive cancer after completing NAC. Detecting this group earlier may support risk-adapted decision-making and timely escalation or modification of therapy. Accordingly, this exploratory study aims to develop a data-driven framework to synthesize conventional ultrasound features into latent phenotypes using model-based latent class analysis (LCA). We investigate whether these holistic sonographic patterns might be associated with pathological non-response to NAC, with a focus on preliminary pattern characterization and hypothesis generation for future validation studies.

## Methods

The study was retrospective and had received approval from the Ethics Committee of Fujian Medical University Union Hospital (Approval No. 2023KY18), with the committee agreeing to waive patient informed consent. The study was conducted in strict accordance with the principles of the Declaration of Helsinki and adheres to the TRIPOD (Transparent Reporting of a Multivariable Prediction Model for Individual Prognosis or Diagnosis) statement for reporting prediction model development (20).

### Patients

This study selected 509 female breast cancer patients who were hospitalized at Fujian Medical University Union Hospital from January 2017 to February 2023. All patients had unilateral primary breast lesions, underwent NAC, and subsequently received surgical resection, with final pathological results obtained. The inclusion criteria were: 1) females aged 18–80 years; 2) suspicious lesions found on imaging examinations and confirmed by biopsy as breast cancer; 3) normal major organ functions and able to tolerate chemotherapy; 4) baseline ultrasound examination performed before NAC with good image quality. Exclusion criteria included: 1) previous anti-tumor treatments; 2) did not complete the established chemotherapy regimen; 3) changed chemotherapy regimen; 4) history of other malignant tumors; 5) pregnancy or lactation; 6) severe organic diseases; 7) factors affecting ultrasound examination results, including poor image quality, significant artifacts or shadowing, post-biopsy changes (hematoma or air), unclear lesion localization, or severe confounding breast pathology.

### Ultrasound image feature extraction

Conventional ultrasound examinations were conducted using calibrated color Doppler ultrasound diagnostic equipment equipped with high-frequency linear array transducers (5–12 MHz or higher), all meeting the technical requirements for breast lesion assessment. The ultrasound image acquisition consistently followed routine standard practice at the center during 2017–2023 (more details in **Supplementary Material**). Two experienced ultrasound physicians (Meilian Zhang and Yi Tang) independently evaluated the image features. We extracted 23 ultrasound image features, which were based on widely recognized descriptors from breast ultrasound reporting systems (BI-RADS) and complemented by additional conventional characteristics deemed clinically relevant for tumor assessment. These features encompassed aspects such as tumor size, morphology, internal echoes, boundary characteristics, blood flow, and changes in the surrounding tissues. If the two physicians disagreed on any feature classification, they discussed the specific image and feature definition and reached a consensus for the final label used in analysis. During feature evaluation, both physicians were completely blinded to patients’ pathological results, NAC treatment information, and any other clinical outcome data. The inter-observer agreement was assessed using Cohen’s kappa coefficient, which demonstrated substantial agreement across the evaluated features (all Kappa values > 0.76). Each feature was standardized and classified accordingly (**Supplementary Material**, **Supplementary Table 1**).

### Postoperative pathological response evaluation

Depending on individual circumstances, patients received chemotherapy regimens including taxanes, anthracyclines, or combination therapies (**Supplementary Material** for details). The pathological response to NAC was assessed using the residual cancer burden (RCB) system or the Miller-Payne (MP) grading system. The RCB system classifies responses into four grades based on tumor size, cellularity, and lymph node involvement, while the MP system grades the reduction in cancer cells into five levels. Based on the evaluation results, patients were categorized into the pathological non-response group (RCB-III or MP Grade 1-2) and the pathological response group (all other cases) (**Supplementary Material**, **Supplementary Table 2**).

### Statistical analysis

Multivariate correspondence analysis (MCA) combined with expert evaluation was first employed to screen the most representative features from breast cancer-related ultrasound image characteristics. We applied three distinct clustering methods—latent class analysis (LCA), K-modes clustering, and hierarchical clustering-each chosen for their strengths in handling categorical data and identifying subtypes. LCA was selected for its model-based probabilistic framework, which is particularly suited for capturing latent heterogeneity (21). K-modes offers a computationally efficient, distance-based partitioning approach specific to categorical data, while hierarchical clustering with Gower distance provides a connectivity-based perspective that reveals natural groupings without pre-specifying cluster count (22, 23). The optimal clustering solution was determined by balancing statistical fit with clinically relevant criteria. In addition to information-theoretic measures and other model fit indicators, we assessed (i) class separation and classification clarity, (ii) clinical interpretability of the resulting categories, and (iii) the strength and consistency of associations with the external clinical outcome of pathological response. This combined strategy is common and recommended in model selection for patient stratification tasks (24).

Consequently, the ultrasound image feature clusters derived from the optimal model were carried forward to investigate their association with pathological non-response using a Firth logistic regression model, a method specifically designed to address the potential bias from the low event rate of pathological non-response in our dataset. To address potential biases from small sample sizes and low event rates, we applied bootstrap resampling (10, 000 iterations) and 10-fold cross-validation to assess model stability and reliability. During bootstrap resampling, only the pathological response group was resampled, while the pathological non-response group remained unchanged, preserving the authenticity of rare events and enhancing model stability. Additionally, subgroup analyses were performed to evaluate the impact of different patient characteristics on the results. Specifically, patients were divided into “high-risk breast cancer factors” (age ≥ 50 years, BMI ≥ 24 kg/m^2^, a family history of breast cancer, and no childbirth history) (25–28) and “non-high-risk factors” groups, and risk differences were iterated through bootstrap resampling (10, 000 iterations) to derive empirical distributions and 95% confidence intervals for each group. Finally, we performed a sensitivity analysis to evaluate the robustness of the findings to the pathological-response definition. Specifically, using the optimal clustering solution, we repeated the clustering after excluding cases assigned by the MP grading system, and then reassessed the associations with pathological non-response using Firth logistic regression with bootstrap resampling (10, 000 iterations) and 10-fold cross-validation, along with the predefined subgroup analyses.

The sample size estimation was based on an expected medium effect size (OR 2.0-3.0), ultimately including 509 patients. Calculations using G*Power software confirmed that this sample size was sufficient to meet the study’s requirements and provided adequate statistical power for subgroup and sensitivity analyses. Moreover, employing bootstrap resampling and cross-validation further ensured the robustness and reproducibility of the study results.

All statistical analyses were performed in R (version 4.4.1) environment, except for LCA, which was conducted using Mplus (version 8.3) (more details in **Supplementary Material**).

## Results

### Baseline characteristics and feature validation

This study included 509 breast cancer patients and analyzed their baseline characteristics before NAC and their pathological response after chemotherapy (**Supplementary Material**, **Supplementary Table 3**). Results showed that 95.68% of the patients responded to chemotherapy, while 4.32% did not. The results of the MCA analysis indicated that the cumulative contribution rate of the first five dimensions reached 32.18%. Although this did not meet the ideal target of 80%, the multivariate structure was sufficiently represented given the complexity and heterogeneity of ultrasound features in our dataset. All 23 ultrasound image variables had contribution rates exceeding the 2% threshold in the first five dimensions, demonstrating their individual informational value (**Supplementary Material**, **Supplementary Figure 1**). Based on these MCA findings, combined with clinical expert review of feature relevance, all 23 variables were retained and entered into subsequent clustering as a comprehensive feature set for identifying distinct ultrasound phenotypes.

### Comparative evaluation of clustering methodologies

The comparative analysis of LCA, K-modes, and hierarchical clustering models demonstrated distinct performance characteristics. For LCA, fit indices supported both two- and three-class solutions with entropy > 0.7 (**Table 1**). While the five-class model showed optimal information criteria, it was rejected due to non-significant LMRT (p = 0.29). External validation confirmed the two-class LCA model achieved statistically significant differentiation in pathological response (p = 0.03) with balanced class distribution, whereas the three-class solution showed no significant association (p = 0.20) (**Table 2**). Conversely, the distance-based partitioning methods were limited by poor internal validity. Both K-modes and hierarchical clustering achieved their maximum average silhouette coefficients at k=2, yet the scores were exceptionally low (0.14 and 0.26 respectively, **Supplementary Material**, **Supplementary Table 4**), indicating weak and unreliable cluster structures. Although K-modes demonstrated a significant association with the pathological outcome (p < 0.001, **Supplementary Material**, **Supplementary Table 5**), this external validity is undermined by its poor internal structure. Hierarchical clustering failed on both fronts, showing no significant association with response (p = 0.76) and producing a clinically uninformative, unstable cluster containing only two cases. Based on superior model fit statistics, meaningful class proportions, and significant external validity, the two-class LCA solution was selected as the most appropriate classification framework for these data.

**Table 1:** Goodness-of-fit indicators for the LCA model.

Category	AIC	BIC	aBIC	Entropy	LMRT	BLRT
One-class	12373.04	12491.55	12402.67	/	/	/
Two-class	12004.00	12245.24	12064.32	0.73	< 0.001	< 0.001
Three-class	11867.25	12231.24	11958.26	0.75	< 0.001	< 0.001
Four-class	11824.34	12311.07	11946.05	0.74	0.64	< 0.001
Five-class	11786.73	12396.20	11939.13	0.77	0.29	< 0.001

AIC, akaike information criterion; BIC, bayesian information criterion; aBIC, adjusted BIC; LMRT, Lo-Mendell-Rubin adjusted likelihood ratio test; BLRT, bootstrap-based likelihood ratio test.

**Table 2:** External validity analysis of the LCA model.

Category	Pathological non-response group	Pathological response group	p-value*
Two-class			
Mode 1	6	251	p=0.03
Mode 2	16	236	
Three-class			
Mode 1	6	125	p=0.20
Mode 2	9	124	
Mode 3	7	238	

*p-value derived from Pearson’s chi-square test.

### Association between latent ultrasound patterns and pathological response

**Figure 1:**
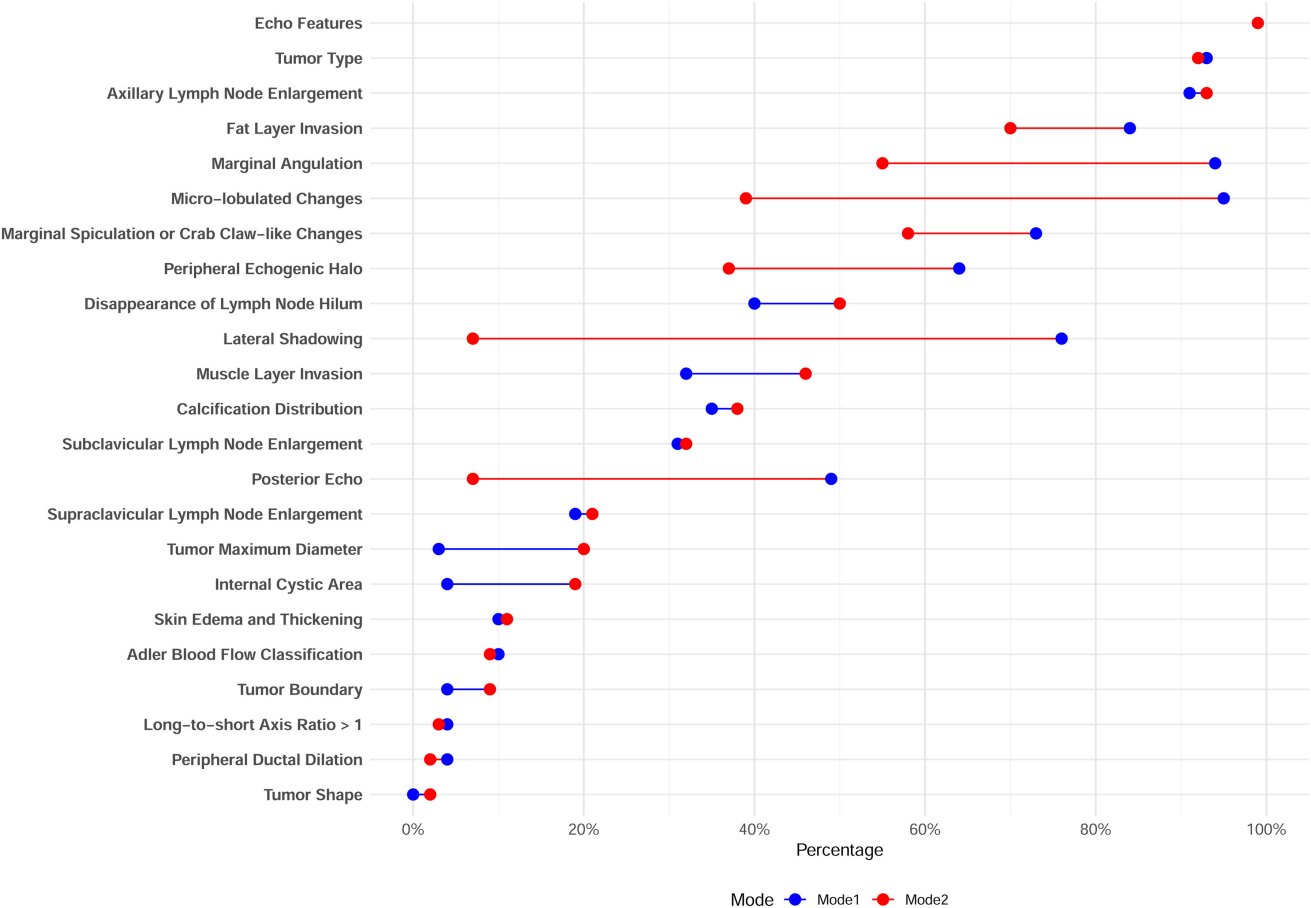
Distribution of bi-classified ultrasound image feature variables using the LCA model (Note: each variable takes the first option). Dots denote ultrasound feature probabilities for two latent modes (blue for Mode 1, red for Mode 2). The connecting line represents the difference in feature probability between the two modes, with longer lines indicating larger differences.

Based on the characteristic distribution of the two-class LCA model (**Figure 1**; **Supplementary Material**, **Supplementary Table 6**), we named the two patterns as Pattern A (50.5%) and Pattern B (49.5%). The main features of Pattern A included: irregular shape (100%), blurred edges (96%), lateral shadowing (76%), posterior echo attenuation (49%), angular margins (94%), spiculated or crab-leg-like margins (73%), micro-lobulated changes (95%), and fat layer invasion (84%). In contrast, Pattern B was characterized by: less lateral shadowing (7%) and posterior echo attenuation (7%), a lower proportion of angular margins (55%) and spiculated margins (58%), fewer micro-lobulated changes (39%), and a lower rate of fat layer invasion (30%).

**Figure 2:**
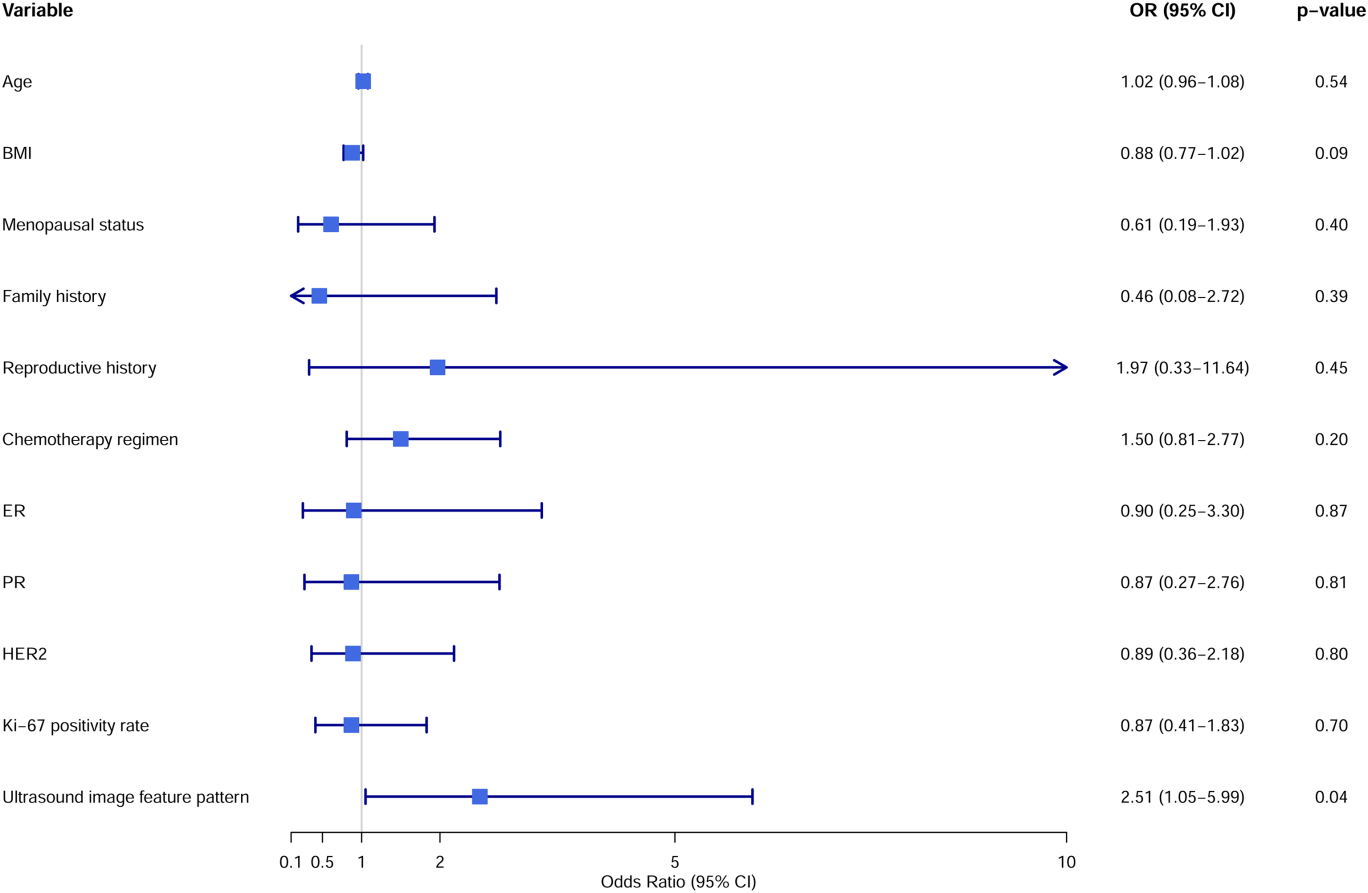
Predictive factors for pathological non-response post-chemotherapy. Forest plot showing the Odds Ratios (OR) and their 95% confidence intervals (CI) for various variables, with squares representing the OR point estimates and horizontal lines indicating the 95% CI.

To explore the association between ultrasound image feature patterns and pathological response to NAC in breast cancer patients, we employed the Firth logistic regression model. First, we calculated the variance inflation factors for all variables, which were all below 2.5, indicating no severe multicollinearity issues. The Firth logistic regression model results showed that Pattern B predicted a 2.51 times higher probability of pathological non-response compared to Pattern A (OR = 2.51, 95% CI: 1.05-5.99, p = 0.04). Other variables such as chemotherapy regimen, age, and menopausal status did not show statistical significance (**Figure 2**). To validate model stability, we performed 10-fold cross-validation and 10, 000 bootstrap iterations. Cross-validation results showed that the OR for ultrasound image feature patterns varied between 1.78 and 2.96 across different folds, with p-values < 0.05 in most folds, supporting its predictive capability (**Table 3**). In the bootstrap analysis (**Table 4**), 7, 493 out of 10, 000 models reached statistical significance (p < 0.05). The ultrasound image feature pattern retained statistical significance in 95% of the significant models, with an average OR of 2.58 (95% CI: 1.07-6.24). In contrast, other variables performed poorly.

**Table 3:** Odds ratios (OR) and 95% confidence intervals (CI) for ultrasound image feature pattern from 10-fold cross-validation.

Fold	OR	CI_lower	CI_upper	p-value
1	2.96	1.17	7.44	0.02
2	2.67	1.06	6.71	0.04
3	2.65	1.04	6.75	0.04
4	2.45	1.03	5.85	0.04
5	2.91	1.15	7.34	0.02
6	2.88	1.13	7.32	0.03
7	2.11	0.87	5.13	0.10
8	2.77	1.11	6.91	0.03
9	2.25	0.93	5.46	0.07
10	1.78	0.71	4.44	0.22

**Table 4:** Odds ratios (OR) and 95% confidence intervals (CI) in the firth logistic regression model (10, 000 bootstrap resamples).

Variable	OR (95% CI) (mean)	% of regression models when variable was significant	Average wald statistic
Age	1.02 (0.96, 1.09)	0.00%	0.00
BMI	0.88 (0.76, 1.02)	19.00%	0.02
Menopausal status	0.62 (0.19, 2.00)	0.00%	0.26
Family history	0.46 (0.07, 2.87)	0.00%	0.78
Childbirth history	2.15 (0.35, 13.30)	0.00%	0.61
Chemotherapy regimen	1.52 (0.81, 2.86)	1.00%	0.18
ER	0.91 (0.24, 3.41)	0.00%	0.05
PR	0.88 (0.27, 2.84)	0.00%	0.05
HER2	0.90 (0.36, 2.23)	0.00%	0.03
Ki-67 positivity rate	0.87 (0.41, 1.86)	0.00%	0.03
Ultrasound Image Feature Pattern	2.58 (1.07, 6.24)	95.00%	0.90

BMI, body mass index.

Subgroup analyses (**Table 5**) demonstrated that patient characteristics moderated the relationship between ultrasound image feature patterns and pathological response to NAC in breast cancer. Although the overall incidence of pathological non-response was low, the magnitude and consistency of the observed effects in subgroup analyses support their clinical relevance. Specifically, for patients categorized into a high-risk group (defined by age ≥ 50 years, BMI ≥ 24 kg/m^2^, family history of breast cancer, no childbirth history), Pattern B was associated with a notable increase in the absolute risk of pathological non-response. This increase was 12.84% (95% CI: 5.74%–23.59%) when taxanes were used, 21.34% (95% CI: 13.63%–28.32%) with anthracyclines, and 21.29% (95% CI: 13.46%–28.48%) with combined chemotherapy regimens. In contrast, the non-high-risk group exhibited smaller risk differences, ranging from 1.12% (95% CI: 0.79%–1.51%) to 3.45% (95% CI: 2.60%–4.47%).

**Table 5:** Impact of chemotherapy regimens on risk differences in NAC response prediction by ultrasound image feature patterns.

Characteristic group	Age	BMI	Family history	Childbirth history	Chemotherapy regimen	Ultrasound image feature pattern risk difference	95% (CI)
High Risk	≥50 years	≥24 kg/m^2^	Present	Absent	Taxane	12.84%	5.74%, 23.59%
High Risk	≥50 years	≥24 kg/m^2^	Present	Absent	Anthracycline	21.34%	13.63%, 28.32%
High Risk	≥50 years	≥24 kg/m^2^	Present	Absent	Combined	21.29%	13.46%, 28.48%
Non-high Risk	<50 years	<24 kg/m^2^	Absent	Present	Taxane	1.12%	0.79%, 1.51%
Non-high Risk	<50 years	<24 kg/m^2^	Absent	Present	Anthracycline	3.45%	2.60%, 4.47%
Non-high Risk	<50 years	<24 kg/m^2^	Absent	Present	Combined	3.36%	2.38%, 4.63%

BMI, body mass index.

### Sensitivity analysis

After excluding the 48 cases assessed by the MP grading system (including 2 cases classified as pathological non-response), we re-ran the LCA and re-clustered the cohort into two patterns; the relative proportions of ultrasound imaging features within Pattern A and Pattern B were comparable to those in the main analysis (**Supplementary Material**, **Supplementary Figure 2**). Pattern B remained a significant predictor of pathological non-response (OR = 2.66, 95% CI: 1.09–6.49, p = 0.03), with statistical significance in the majority of cross-validation folds and in nearly all bootstrap resamples, whereas other variables showed no clear association (**Supplementary Material**, **Supplementary Tables 7**-**S9**). Moreover, the pattern-related risk difference across chemotherapy regimens persisted in both high- and non-high-risk groups, with larger risk differences observed in the high-risk context (**Supplementary Material**, **Supplementary Table 10**).

## Discussion

In this exploratory study, we sought to determine if a holistic analysis of conventional ultrasound features could identify patterns potentially associated with NAC response. A critical preliminary step was comparing a model-based approach (LCA) with distance-based algorithms (K-modes and hierarchical clustering). LCA proved to be a suitable method for this cohort, identifying two robust and clinically relevant patterns. Although the MCA cumulative contribution rate of 32.18% is relatively low, this should be interpreted in the context of LCA modeling. A limited explained contribution in MCA suggests that the categorical ultrasound descriptors form a distributed and multi-faceted structure rather than being summarized by a small number of dominant dimensions. This does not preclude identification of latent classes; accordingly, our LCA solution was evaluated using model fit and associations with the external clinical outcome of pathological response. For the purpose of interpretation, we designated these as Pattern A and Pattern B, while acknowledging that these terms represent composites of sonographic features rather than confirmed biological entities. The core contribution lies in generating hypotheses about their potential stratified predictive power; Pattern B was observed to be associated with pathological non-response to NAC, especially within clinically defined high-risk patient subgroups.

Predicting NAC efficacy is a pivotal goal in breast cancer management, with pCR being a primary endpoint strongly associated with improved long-term survival, particularly in aggressive subtypes like triple-negative and HER2-positive breast cancer (29). Several computational approaches have shown promise in this domain, such as a deep learning radiomics nomogram based on ultrasound imaging (AUC = 0.94) (30) and a combined ultrasound-clinicopathological nomogram (C-index = 0.79) (31). However, these advanced models often require complex feature extraction or multi-modal data not universally accessible in clinical settings. Conventional ultrasound alone demonstrates more modest utility, with limited sensitivity (60.8%) and specificity (78.0%) for pCR prediction and variable accuracy across molecular subtypes (18). This indicates a potential ceiling for conventional ultrasound in detecting the subtle characteristics of patients achieving pCR—the “best of the good”—particularly given the generally high pathological response rates in breast cancer (32, 33). This limitation redirected our research focus toward identifying patients with pathological non-response—the “worst of the good”. These patients typically have poorer prognoses and may present more distinct imaging patterns detectable through conventional ultrasound. Our findings are consistent with this hypothesis. We identified Pattern B associated with a higher likelihood of non-response. The use of routinely collected ultrasound features provides a foundation for future validation studies. Moreover, our classification approach was designed with features intended to facilitate eventual clinical workflow. Given ongoing debates about the direct translation of pCR rates to long-term survival benefits at the trial level (34), our results underscore the potential clinical relevance of an alternative strategy focused on identifying non-responders, which could provide a valuable opportunity to optimize treatment pathways for this high-risk population. However, it is important to acknowledge that the relatively low pathological non-response rate in our cohort limited the feasibility of robust calibration plot analysis. Furthermore, a comprehensive assessment of net clinical benefit through decision curve analysis would require prospective data on outcomes under alternative treatment strategies, which were beyond the scope of this retrospective study.

The efficacy of NAC varies significantly across different molecular subtypes of breast cancer. Specifically, in HER2-positive patients, NAC significantly increases the pCR rate and improves long-term prognosis. Research indicates that systemic treatment strategies for HER2-positive breast cancer are continually evolving, making stratified risk assessments essential for selecting the appropriate treatment (35). TNBC is known for its high aggressiveness and poor prognosis, and it has traditionally relied primarily on chemotherapy. Recent studies have found that adding carboplatin to the conventional anthracycline/taxane-based NAC regimen can increase the pCR rate and improve long-term outcomes (36). Additionally, the application of immune checkpoint inhibitors in high-risk TNBC patients has shown potential in improving the pCR rate (36). For low-risk patients (e.g., tumors smaller than 20 mm and lymph node-negative), NAC may not be necessary, thereby avoiding unnecessary treatment (35). This established clinical heterogeneity provided the rationale for our subgroup analysis. We hypothesized that the association between our candidate sonographic patterns and treatment response would likewise differ across subgroups. Our findings suggest that the association between Pattern B and pathological non-response was most pronounced in the clinically defined high-risk patient group. This observation is consistent with the principle of subtype-dependent NAC efficacy. It generates the hypothesis that the potential utility of these sonographic signatures may be greatest in specific high-risk contexts. It is crucial to acknowledge that tumor molecular subtype (e.g., TNBC vs. luminal) represents a significant potential confounder, as these intrinsic biological differences not only influence tumor morphology visible on ultrasound but also dictate the likelihood of NAC response. The varying proportions of these subtypes across patient cohorts could therefore influence observed associations. However, using chemotherapy regimen as a proxy for underlying molecular subtypes in our study is indirect. Chemotherapy selection reflects complex clinical decisions rather than precise molecular stratification alone, which can obscure direct links between ultrasound patterns and specific molecular biology. Future research needs directly integrate detailed molecular pathology with sonographic analysis to clarify these relationships and enhance the clinical interpretability of such imaging tools.

Our descriptive label for sonographic patterns does not equate to a direct measure of biological tumor activity (37). Our statistical finding prompts several hypotheses regarding its potential biological underpinnings, especially since ultrasound can reflect aspects of tumor heterogeneity and the microenvironment (38, 39). For instance, Pattern B might signify a more slowly proliferating phenotype, which would be inherently less susceptible to chemotherapies that target rapidly dividing cells. Alternatively, these features might correspond to stromal-rich tumors, where a dense extracellular matrix could physically impede drug delivery. It is also plausible that tumors presenting with this pattern harbor intrinsically drug-resistant cell subpopulations or that specific microenvironmental factors, such as hypoxia or an acidic pH, are reflected in the imaging and contribute to resistance. Finally, structural characteristics suggested by the pattern, like fibrosis or vascular architecture, may influence drug penetration. Unraveling the complex relationship between sonographic appearance and tumor biology is beyond the scope of this study, and dedicated future research integrating imaging with analyses of molecular mechanisms is essential to test these hypotheses.

Although this study has yielded some valuable findings, there are several limitations to note: 1) This study is a single-center retrospective analysis. While it is useful for exploratory assessment and hypothesis generation, the reliability and generalizability of the identified ultrasound phenotypes may be influenced by institutional practice, ultrasound acquisition routines, and operator-dependent feature interpretation. Future work will therefore focus on external evaluation in independent multicenter cohorts and the use of standardized reporting frameworks and descriptors (e.g., BI-RADS terminology) to harmonize feature definition and interpretation. We will also consider calibration procedures for image readers across sites prior to validation to improve consistency. 2) The exclusion of cases with inadequate ultrasound image quality may introduce spectrum bias, as patients with more accessible lesions or less complex anatomy—which could be associated with lower tumor aggressiveness—may be preferentially retained, potentially affecting the representativeness of our findings.3) This study focused only on the pathological response to NAC as an intermediate endpoint, lacking evaluation of long-term outcomes such as patient survival. This limits the long-term application value of the study results in guiding clinical decision-making. 4) The study did not assess the potential impact of patients’ psychological states (such as anxiety and depression) on treatment response. This could influence patients’ tolerance to chemotherapy and their response to treatment, thereby affecting the study results. 5) The study only considered the family history of breast cancer without delving into the potential impact of patients’ genetic backgrounds (such as gene mutations) on chemotherapy response.

## Conclusion

This study suggests an association between conventional ultrasound feature patterns and pathological response to NAC, particularly in high-risk patients. These hypothesis-generating findings warrant external validation to evaluate whether the identified patterns can generalize across settings and contribute to non-invasive risk stratification in future work.

## Data Availability

The original contributions presented in the study are included in the article/**Supplementary Material**. Further inquiries can be directed to the corresponding authors.
