# Supplementary material for: Latent class analysis of conventional ultrasound features: a novel approach to predicting non-response to neoadjuvant chemotherapy in breast cancer
Source: Front Oncol. 2026 May 7;16:1813724. doi: 10.3389/fonc.2026.1813724 (PMC13189786; doi:10.3389/fonc.2026.1813724)
Supplement: Supplementary file 1 [file DataSheet1.doc]

**Latent Class Analysis of Conventional Ultrasound Features: A Novel Approach to Predicting Non-Response to Neoadjuvant Chemotherapy in Breast Cancer**

Running title：Ultrasound Features Predict NAC Non-Response in Breast Cancer

Mei-Lian Zhang^1*^, Yi Tang^1*^, Cheng-Hua Kong^2^, Li Sun^2^, Xing-Qing Li^2^,

Yu Zhou^2#^, Zhi-Kui Chen^1#^

^1^Department of Ultrasound, Fujian Medical University Union Hospital, Fuzhou, China

^2^Department of Clinical Pharmacy and Pharmacy Administration, School of Pharmacy, Fujian Medical University, Fuzhou, China

*Contributed equally

^#^Corresponding author: Zhikui Chen, zhikui_chen@fjmu.edu.cn and Yu Zhou, [cfjsczy@fjmu.edu.cn](mailto:cfjsczy@fjmu.edu.cn)

**Supplement Materials**

**Materials and Methods**

**Classification of Chemotherapy Regimens**

The classification of chemotherapy regimens is primarily based on the type of drugs and their mechanisms of action. The specific regimens and their classifications are as follows:

A. Taxane-based Regimens: These regimens are based on paclitaxel and docetaxel, which primarily inhibit microtubule formation, thereby preventing cancer cell division.

1. P regimen: Paclitaxel
2. PX regimen: Paclitaxel + Capecitabine
3. PCb regimen: Paclitaxel + Carboplatin
4. T regimen: Docetaxel
5. TC regimen: Docetaxel + Cyclophosphamide
6. TCb regimen: Docetaxel + Carboplatin
7. TP regimen: Docetaxel + Cisplatin

B. Anthracycline-based Regimens: These regimens are based on doxorubicin and epirubicin, which work by interfering with DNA synthesis and repair, affecting cancer cell replication.

1. AC regimen: Doxorubicin + Cyclophosphamide
2. EC regimen: Epirubicin + Cyclophosphamide

C. Combined Regimens: These combine different types of chemotherapy drugs, including taxanes, anthracyclines, and HER2-targeted agents (such as trastuzumab and pertuzumab), to enhance efficacy, particularly in the treatment of HER2-positive breast cancer.

1. AC-T regimen: Doxorubicin + Cyclophosphamide + Docetaxel
2. EC-T regimen: Epirubicin + Cyclophosphamide + Docetaxel
3. PH regimen: Paclitaxel + Trastuzumab
4. TH regimen: Docetaxel + Trastuzumab
5. TH + Pyrotinib regimen: Docetaxel + Trastuzumab + Pyrotinib
6. TCbH regimen: Docetaxel + Carboplatin + Trastuzumab
7. TCbH+ Pyrotinib regimen: Docetaxel + Carboplatin + Trastuzumab + Pyrotinib
8. TCbHP regimen: Docetaxel + Carboplatin + Trastuzumab + Pertuzumab
9. PCbHP regimen: Paclitaxel + Carboplatin + Trastuzumab + Pertuzumab
10. TCH regimen: Docetaxel + Cyclophosphamide + Trastuzumab
11. THP regimen: Docetaxel + Trastuzumab + Pertuzumab

**Ultrasound Image Acquisition Protocol**

For equipment and parameter settings, a linear-array high-frequency transducer (center frequency 5-12 MHz or higher) was used with depth commonly set to 3-4 cm to keep the lesion in the center of the imaging field, and the focus was positioned at the lesion. Images included breast tissue and the posterior pectoral muscle; the maximum imaging depth was adjusted to display the pleura while avoiding excessive lung inclusion. For larger lesions, wide-field imaging was applied. No special preparation was required before examination. Patients were examined in the supine position; both breasts and the axillary regions were exposed, with both arms raised naturally and placed on the sides of the head. If breasts were larger or more pendulous, the patient was gently supported to adjust so that the breast was balanced. Scanning covered the whole breast bilaterally, bilateral axillae, and the supraclavicular and infraclavicular fossa areas. The scanning order was left to right, using radial, anti-radial, longitudinal, transverse, and oblique planes to ensure comprehensive coverage without omission. Standard image recording included: for normal breast, one image from the slice showing the maximum glandular tissue on each side; for detected lesions, the maximum long-axis slice, the slice perpendicular to it, the slice with the richest Doppler signal, and the corresponding axillary lymph node maximum long-axis slice and blood-flow slice on the involved side. Probe position and direction were annotated using surface markers. For color Doppler, the sampling box included the lesion and at least a 1 cm surrounding margin, with a velocity scale typically set to 3–5 cm/s to avoid pressure-related flow artifacts. During data collection, only ultrasound images meeting predefined quality criteria were included for subsequent feature assessment.

**Statistical Analysis**

**Multiple Correspondence Analysis (MCA)**

We adopted a method combining MCA and expert evaluation to filter the most representative and informative features from a multitude of breast cancer ultrasound image variables. The reason for choosing MCA is its suitability for handling categorical variables, primarily binary, and its effectiveness in analyzing complex relationships among multiple variables. The analysis included steps such as data preparation, MCA analysis, contribution rate calculation, key dimension determination, and variable selection. We set a target cumulative contribution rate of 80% and a contribution rate threshold of 2% to identify key dimensions and screen important variables. Finally, by integrating the MCA statistical results with clinical expert opinions, we determined the most representative and clinically significant ultrasound image variables.

**Latent Class Analysis (LCA)**

LCA is a statistical method used to identify latent subgroups or categories within observed data(1). This method assumes that there are latent categories that can explain the relationships between observed variables, making it particularly suitable for categorical and discrete data. It can identify groups of individuals with similar characteristics based on patterns across multiple observed variables. In this study, LCA was used to simultaneously consider multiple ultrasound image features, identify latent subtypes, and associate the clustering results with the pathological responsiveness following neoadjuvant chemotherapy (NAC).

The LCA models were constructed and fitted using Mplus version 8.3 software. Manifest variables included in the analysis were determined from the previous MCA step. Model fit was primarily evaluated based on the following criteria(2): 1) Information criteria: Akaike Information Criterion (AIC), Bayesian Information Criterion (BIC), and sample-size adjusted BIC (aBIC). Lower values of these criteria indicate better model fit. 2) Entropy: This index evaluates the accuracy of classification, with a range from 0 to 1. Higher values, closer to 1, indicate more precise class assignments. 3) Likelihood ratio tests: These include the Lo-Mendell-Rubin adjusted likelihood ratio test (LMRT) and the bootstrap-based likelihood ratio test (BLRT). p-values less than 0.05 for these tests suggest that the k-class model fits significantly better than the k-1 class model.

Following the latent class analysis, we analyzed the relationship between the identified latent classes of ultrasound image features and the pathological responsiveness to NAC to assess the external validity of the model classification. Differences in pathological response proportions between the classes were tested using Pearson's chi-square test (or Fisher's exact test), with statistical significance set at p < 0.05.

**K-modes Clustering**

K-modes clustering was employed as a non-hierarchical, distance-based partitioning method to provide a comparative perspective(3). The number of clusters (k) was predetermined to range from 2 to 5. For each k value in this range, the algorithm was executed 50 times with random initializations to avoid local optima, and the solution with the lowest total within-cluster dissimilarity was retained as the optimal model for that specific k. The specific seed that generated this optimal run was recorded, ensuring that the final model is fully reproducible. The final selection of the optimal k was guided by the highest average silhouette coefficient, a metric used to assess the internal validity of the cluster structure by measuring both cohesion and separation(4). This metric was calculated using a Gower distance matrix, which is specifically appropriate for the categorical nature of our ultrasound features. The coefficient ranges from -1 to 1; a value close to 1 indicates that objects are well-matched to their own cluster and distinct from neighboring clusters, signifying a strong and appropriate structure. A value near zero suggests overlapping clusters, while a negative value indicates that an object may have been assigned to the incorrect cluster. It has been argued that average silhouette coefficients greater than 0.70 indicate a strong structure, those below 0.50 indicate a weak structure, and those < 0.25 indicate little evidence for any reliable structure(5, 6). After identifying the optimal K-modes solution, its external validity was formally assessed by using Pearson's chi-square test (or Fisher's exact test) to analyze for significant differences in pathological response rates across the derived clusters.

**Hierarchical Clustering**

Hierarchical clustering was performed to explore the natural, connectivity-based groupings within the data(7). Given the categorical nature of the ultrasound features, Gower distance was employed as the dissimilarity metric. This was used in conjunction with the average linkage method to construct the cluster dendrogram. The number of clusters (k) was selected through a structured evaluation framework, assessing candidate solutions from k=2 to k=5. As previously described, the final selection of the optimal k was guided by the highest average silhouette coefficient, calculated from the Gower distance matrix, which reflects the quality of cluster cohesion and separation. Once the optimal k was determined, the external validity of the resulting clusters was assessed by using Pearson's chi-square test (or Fisher's exact test) to analyze for significant differences in pathological response rates across the groups.

**Penalized Logistic Regression Analysis and Internal Validation**

Based on the optimal model, we performed penalized logistic regression analysis (Firth's penalized logistic regression) using the latent classes of ultrasound image feature patterns as independent variables. Covariates included age, BMI, menopausal status, family history of breast cancer, childbirth history, chemotherapy regimen, and key breast cancer biomarkers (ER, PR, HER2, and Ki-67). The dependent variable was the pathological response status following NAC. Prior to applying the logistic regression model, we used a conservative variance inflation factor (VIF) threshold of 2.5 to exclude covariates exhibiting collinearity, ensuring the stability and validity of the model(8). By estimating the odds ratios (OR) and their standard errors (SE), we derived the risk differences between different ultrasound image feature pattern groups and their 95% confidence intervals (CI). The use of penalized logistic regression helps to address potential biases in situations with low event rates, offering advantages over traditional logistic regression models(9). Following the Firth logistic regression model, a 10-fold cross-validation was performed to assess the stability and reliability of the model, quantify the uncertainty of predictors, and enhance the reproducibility of the study results.

To address the issue of small sample sizes and reduce the result bias due to sample specificity, we further employed bootstrap resampling to evaluate model stability and reliability(8). Specifically, for each iteration, we performed resampling with replacement from the pathological response group and combined it with the original pathological non-response group to form a subset. Penalized logistic regression analysis was conducted in each iteration, recording each model's Nagelkerke R² and p-values, as well as the β coefficients, Wald statistics, p-values, odds ratios (OR), and their 95% confidence intervals for each predictor. After fitting the model 10,000 times, we performed a summary analysis of the results. Overall model significance was first adjusted using Bonferroni correction, followed by Bonferroni correction for individual predictor significance, with a corrected p-value of 0.05. We only retained predictors that were statistically significant in more than 60% of the significant models as the final robust and reproducible results.

**Sensitivity Analysis**

Based on the aforementioned bootstrap resampling, we conducted a sensitivity analysis to evaluate the impact of different patient characteristics on the relationship between ultrasound image feature patterns and pathological response to NAC in breast cancer. The specific steps were as follows:

1) Grouping Patients: Patients were divided into two groups:

- High-Risk Factors for Breast Cancer: Patients with characteristics such as age ≥ 50 years, BMI ≥ 24, a family history of breast cancer, and no childbirth history(10-13).

- Non-High-Risk Factors for Breast Cancer: Patients who did not meet the above high-risk characteristics.

2) Bootstrap Iterations: In each bootstrap iteration, we calculated the risk differences in predicting pathological non-response among different ultrasound image feature patterns for each chemotherapy regimen combination, separately for the two patient groups.

3) Summary of Results: We summarized the risk difference results from 10,000 iterations to obtain the empirical distribution of risk differences for each group. From the empirical distribution, we extracted the 95% confidence intervals as the final risk difference estimates and their uncertainties.

All statistical analyses were performed in R (version 4.4.1) environment, except for LCA, which was conducted using Mplus (version 8.3).

**References**

1. Collins LM, Lanza ST. Latent class and latent transition analysis: With applications in the social, behavioral, and health sciences: John Wiley & Sons; 2009.

2. Ciesinski NK, Drabick DAG, McCloskey MS. A latent class analysis of intermittent explosive disorder symptoms. Journal of affective disorders. 2022;302:367-75.

3. Huang Z. Extensions to the k-Means Algorithm for Clustering Large Data Sets with Categorical Values. Data Mining and Knowledge Discovery. 1998;2(3):283-304.

4. Rousseeuw PJ. Silhouettes: A graphical aid to the interpretation and validation of cluster analysis. Journal of Computational and Applied Mathematics. 1987;20:53-65.

5. Kaufman L, Rousseeuw PJ. Finding groups in data: an introduction to cluster analysis: John Wiley & Sons; 2009.

6. Cane J, O'Connor D, Michie S. Validation of the theoretical domains framework for use in behaviour change and implementation research. Implement Sci. 2012;7:37.

7. Oti E, Olusola M. Overview of agglomerative hierarchical clustering methods. British Journal of Computer, Networking and Information Technology. 2024;7(2):14-23.

8. Janiri D, Doucet GE, Pompili M, Sani G, Luna B, Brent DA, et al. Risk and protective factors for childhood suicidality: a US population-based study. The lancet Psychiatry. 2020;7(4):317-26.

9. Fischer U, Koga M, Strbian D, Branca M, Abend S, Trelle S, et al. Early versus Later Anticoagulation for Stroke with Atrial Fibrillation. The New England journal of medicine. 2023;388(26):2411-21.

10. Yaghjyan L, Austin-Datta RJ, Oh H, Heng YJ, Vellal AD, Sirinukunwattana K, et al. Associations of reproductive breast cancer risk factors with breast tissue composition. Breast cancer research : BCR. 2021;23(1):70.

11. Renehan AG, Tyson M, Egger M, Heller RF, Zwahlen M. Body-mass index and incidence of cancer: a systematic review and meta-analysis of prospective observational studies. Lancet. 2008;371(9612):569-78.

12. Łukasiewicz S, Czeczelewski M, Forma A, Baj J, Sitarz R, Stanisławek A. Breast Cancer-Epidemiology, Risk Factors, Classification, Prognostic Markers, and Current Treatment Strategies-An Updated Review. Cancers. 2021;13(17):4287.

13. Wang H, MacInnis RJ, Li S. Family history and breast cancer risk for Asian women: a systematic review and meta-analysis. BMC medicine. 2023;21(1):239.

**Table S1** Extraction and Statistical Classification of Conventional Ultrasound Image Feature Variables

| **No.** | **Variable** | **Description** | **Classification** | **Kappa value** |
| --- | --- | --- | --- | --- |
| 1 | Tumor Maximum Diameter | Measured using the ultrasound machine’s built-in measurement tool on three different planes (transverse, sagittal, and coronal), taking the maximum value in millimeters (mm). | ≤ 2 cm (Value 1); > 2 cm and ≤ 5 cm (Value 2); > 5 cm (Value 3) | 0.88 |
| 2 | Tumor Type | Observing the shape and boundary characteristics of the mass. | Mass (Value 1); Non-mass (Value 2) | 0.85 |
| 3 | Echo Features | Observing the internal echo of the mass. | Hypoechoic (Value 1); Hyperechoic (Value 2) | 0.82 |
| 4 | Tumor Shape | Assessing the overall morphology of the mass. | Regular (Value 1); Irregular (Value 2) | 0.86 |
| 5 | Tumor Boundary | Observing the clarity between the mass and surrounding tissues. | Clear (Value 1); Fuzzy (Value 2) | 0.84 |
| 6 | Lateral Shadowing | Observing whether there are shadows on both sides of the mass. | Present (Value 1); Absent (Value 2) | 0.79 |
| 7 | Posterior Echo | Assessing changes in the echo behind the mass. | Attenuation (Value 1); No attenuation or enhancement (Value 2) | 0.77 |
| 8 | Calcification Distribution | Observing the presence and distribution of calcifications within the mass. | No calcifications (Value 0); Clustered (Value 1); Scattered (Value 2); Clustered + Scattered (Value 3) | 0.81 |
| 9 | Adler Blood Flow Classification | After 2D ultrasound (2DUS) exploration, using color Doppler flow imaging (CDFI) to observe the blood flow within and around the tumor. | Grade 0: No blood flow detected (Value 0); Grade I: Minimal blood flow (Value 1); Grade II: Moderate blood flow (Value 2); Grade III: Rich blood flow (Value 3) | 0.83 |
| 10 | Marginal Angulation | Observing whether there are sharp angles at the edges of the mass. | Present (Value 1); Absent (Value 2) | 0.80 |
| 11 | Marginal Spiculation or Crab Claw-like Changes | Observing whether there are spiculations or crab claw-like protrusions at the edges of the mass. | Present (Value 1); Absent (Value 2) | 0.87 |
| 12 | Peripheral Echogenic Halo | Observing the presence of an echogenic halo around the mass. | Present (Value 1); Absent (Value 2) | 0.78 |
| 13 | Micro-lobulated Changes | Observing the presence of micro-lobulations at the edges of the mass. | Present (Value 1); Absent (Value 2) | 0.89 |
| 14 | Internal Cystic Area | Observing whether there are cystic areas within the mass. | Present (Value 1); Absent (Value 2) | 0.90 |
| 15 | Peripheral Ductal Dilation | Observing whether there is ductal dilation around the mass. | Present (Value 1); Absent (Value 2) | 0.79 |
| 16 | Skin Edema and Thickening | Observing whether there is skin edema and thickening. | Present (Value 1); Absent (Value 2) | 0.81 |
| 17 | Axillary Lymph Node Enlargement | Checking for enlarged lymph nodes in the axillary region. | Present (Value 1); Absent (Value 2) | 0.85 |
| 18 | Disappearance of Lymph Node Hilum | Observing the internal structure of enlarged lymph nodes. | Disappeared (Value 1); Not disappeared (Value 2) | 0.83 |
| 19 | Fat Layer Invasion | Observing whether the tumor invades the subcutaneous fat layer. | Present (Value 1); Absent (Value 2) | 0.86 |
| 20 | Muscle Layer Invasion | Observing whether the tumor invades the pectoral muscle. | Present (Value 1); Absent (Value 2) | 0.88 |
| 21 | Long-to-short Axis Ratio > 1 | Measuring the maximum diameters in the vertical and horizontal directions and calculating the ratio. | >1 (Value 1); ≤1 (Value 2) | 0.84 |
| 22 | Subclavicular Lymph Node Enlargement | Checking for enlarged lymph nodes in the subclavicular region. | Present (Value 1); Absent (Value 2) | 0.77 |
| 23 | Supraclavicular Lymph Node Enlargement | Checking for enlarged lymph nodes in the supraclavicular region. | Present (Value 1); Absent (Value 2) | 0.78 |

**Table S2** Mapping of Pathological Response Grading Systems

| **System** | **Description / Grade** | **Corresponding Study Category** |
| --- | --- | --- |
| Miller and Payne System | | |
| Grade 1 | No change or some alteration to individual malignant cells but no reduction in overall cellularity. | Pathological Non-response |
| Grade 2 | A minor loss of tumor cells but overall cellularity still high; up to 30% loss. | Pathological Non-response |
| Grade 3 | Between an estimated 30% and 90% reduction in tumor cells. | Pathological Response |
| Grade 4 | A marked disappearance of tumor cells such that only small clusters or widely dispersed individual cells remain; more than 90% loss of tumor cells. | Pathological Response |
| Grade 5 | No malignant cells identifiable in sections from the site of the tumor; only vascular fibroelastic stroma remains often containing macrophages. However, ductal carcinoma in situ may be present. | Pathological Response |
| Residual Cancer Burden System | | |
| RCB-0 | Pathologic complete response | Pathological Response |
| RCB-I | Minimal residual disease | Pathological Response |
| RCB-II | Moderate residual disease | Pathological Response |
| RCB-III | Extensive residual disease | Pathological Non-response |

**Table S3** Baseline Characteristics of Breast Cancer Patients Before NAC and Pathological Response Post-Chemotherapy (n=509)

| **Variable** | **Category** | **Frequency** | **Percentage (%)** |
| --- | --- | --- | --- |
| Age | < 50 years | 274 | 53.83 |
|  | ≥ 50 years | 235 | 46.17 |
| BMI | BMI < 24 kg/m^2^ | 267 | 52.46 |
|  | BMI ≥ 24 kg/m^2^ | 242 | 47.54 |
| Menopausal status | Present | 206 | 40.47 |
|  | Absent | 303 | 59.53 |
| Family history | Present | 13 | 2.55 |
|  | Absent | 496 | 97.45 |
| Childbirth history | Present | 487 | 95.68 |
|  | Absent | 22 | 4.32 |
| Chemotherapy regimen | Taxane | 128 | 25.15 |
|  | Anthracycline | 283 | 55.60 |
|  | Combined | 98 | 19.25 |
| Pathological Response | Response | 487 | 95.68 |
|  | Non-response | 22 | 4.32 |
| **Molecular markers of breast cancer** | | | |
| ER | Positive | 386 | 75.83 |
|  | Negative | 123 | 24.17 |
| PR | Positive | 311 | 61.10 |
|  | Negative | 198 | 38.90 |
| HER2 | Positive | 335 | 65.82 |
|  | Negative | 172 | 33.79 |
|  | Uncertainty | 2 | 0.39 |
| Ki-67 positivity rate | ≤30% | 210 | 41.26 |
|  | ＞30% | 293 | 57.56 |
|  | Uncertainty | 6 | 1.18 |
| **Ultrasonic image feature** | | | |
| Tumor Maximum Diameter | ≤ 2 cm | 58 | 11.39 |
|  | > 2 cm and ≤ 5 cm | 359 | 70.53 |
|  | > 5 cm | 92 | 18.07 |
| Tumor Type | Mass-type | 472 | 92.73 |
|  | Non-mass-type | 37 | 7.27 |
| Echo Features | Hypoechoic | 503 | 98.82 |
|  | Hyperechoic | 6 | 1.18 |
| Tumor Shape | Regular | 5 | 0.98 |
|  | Irregular | 504 | 99.02 |
| Tumor Boundary | Clear | 34 | 6.68 |
|  | Indistinct | 475 | 93.32 |
| Lateral Shadowing | Present | 212 | 41.65 |
|  | Absent | 297 | 58.35 |
| Posterior Echo | Attenuation | 143 | 28.09 |
|  | No attenuation or enhancement | 366 | 71.91 |
| Calcification Distribution | No calcification | 184 | 36.15 |
|  | Clustered | 62 | 12.18 |
|  | Scattered | 160 | 31.43 |
|  | Clustered + scattered | 103 | 20.24 |
| Adler Blood Flow Classification | Grade 0 | 49 | 9.63 |
|  | Grade 1 | 163 | 32.02 |
|  | Grade 2 | 170 | 33.40 |
|  | Grade 3 | 127 | 24.95 |
| Marginal Angulation | Present | 380 | 74.66 |
|  | Absent | 129 | 25.34 |
| Marginal Spiculation or Crab Claw-like Changes | Present | 335 | 65.82 |
|  | Absent | 174 | 34.18 |
| Peripheral Echogenic Halo | Present | 256 | 50.29 |
|  | Absent | 253 | 49.71 |
| Micro-lobulated Changes | Present | 342 | 67.19 |
|  | Absent | 167 | 32.81 |
| Internal Cystic Area | Present | 56 | 11.00 |
|  | Absent | 453 | 89.00 |
| Peripheral Ductal Dilation | Present | 16 | 3.14 |
|  | Absent | 493 | 96.86 |
| Skin Edema and Thickening | Present | 51 | 10.02 |
|  | Absent | 458 | 89.98 |
| Axillary Lymph Node Enlargement | Present | 469 | 92.14 |
|  | Absent | 40 | 7.86 |
| Disappearance of Lymph Node Hilum | Present | 229 | 44.99 |
|  | Absent | 280 | 55.01 |
| Fat Layer Invasion | Present | 392 | 77.01 |
|  | Absent | 117 | 22.99 |
| Muscle Layer Invasion | Present | 197 | 38.70 |
|  | Absent | 312 | 61.30 |
| Long-to-short Axis Ratio > 1 | Present | 17 | 3.34 |
|  | Absent | 492 | 96.66 |
| Subclavicular Lymph Node Enlargement | Present | 159 | 31.24 |
|  | Absent | 350 | 68.76 |
| Supraclavicular Lymph Node Enlargement | Present | 102 | 20.04 |
|  | Absent | 407 | 79.96 |

**Table S4** Average Silhouette Coefficients for the K-modes and Hierarchical Clustering Models

|  | K-modes clustering | Hierarchical clustering |
| --- | --- | --- |
| Category | Average silhouette coefficient | Average silhouette coefficient |
| Two-class | 0.14 | 0.26 |
| Three-class | 0.12 | 0.18 |
| Four-class | 0.10 | 0.12 |
| Five-class | 0.10 | 0.08 |

**Table S5** External Validity Analysis of Binary Classifications from the K-modes and Hierarchical Clustering Models

|  | Pathological non-response group | Pathological  response group | p-value* |
| --- | --- | --- | --- |
| K-modes clustering | | | |
| Mode 1 | 18 | 190 | p < 0.001 |
| Mode 2 | 4 | 297 |
| Hierarchical clustering | | | |
| Mode 1 | 22 | 485 | p = 0.76 |
| Mode 2 | 0 | 2 |

*For K-modes clustering: p-value derived from Pearson's Chi-square test; For hierarchical clustering: p-value derived from Fisher's exact test.

**Table S6** Distribution of Bi-classified Ultrasound Image Feature Variables Using LCA (n=509)

| **Variable** | **Category** | **Mode 1（%）**  **n=257** | **Mode 2（%）**  **n=252** |
| --- | --- | --- | --- |
| Tumor Maximum Diameter | ≤ 2 cm | 0.03 | 0.20 |
|  | > 2 cm and ≤ 5 cm | 0.85 | 0.56 |
|  | > 5 cm | 0.12 | 0.25 |
| Tumor Type | Mass-type | 0.93 | 0.92 |
|  | Non-mass-type | 0.07 | 0.08 |
| Echo Features | Hypoechoic | 0.99 | 0.99 |
|  | Hyperechoic | 0.01 | 0.02 |
| Tumor Shape | Regular | 0.00 | 0.02 |
|  | Irregular | 1.00 | 0.98 |
| Tumor Boundary | Clear | 0.04 | 0.09 |
|  | Indistinct | 0.96 | 0.91 |
| Lateral Shadowing | Present | 0.76 | 0.07 |
|  | Absent | 0.24 | 0.93 |
| Posterior Echo | Attenuation | 0.49 | 0.07 |
|  | No attenuation or enhancement | 0.51 | 0.93 |
| Calcification Distribution | No calcification | 0.35 | 0.38 |
|  | Clustered | 0.16 | 0.08 |
|  | Scattered | 0.26 | 0.37 |
|  | Clustered + scattered | 0.23 | 0.17 |
| Adler Blood Flow Classification | Grade 0 | 0.10 | 0.09 |
|  | Grade 1 | 0.38 | 0.26 |
|  | Grade 2 | 0.32 | 0.35 |
|  | Grade 3 | 0.20 | 0.31 |
| Marginal Angulation | Present | 0.94 | 0.55 |
|  | Absent | 0.06 | 0.45 |
| Marginal Spiculation or Crab Claw-like Changes | Present | 0.73 | 0.58 |
|  | Absent | 0.27 | 0.42 |
| Peripheral Echogenic Halo | Present | 0.64 | 0.37 |
|  | Absent | 0.36 | 0.63 |
| Micro-lobulated Changes | Present | 0.95 | 0.39 |
|  | Absent | 0.05 | 0.61 |
| Internal Cystic Area | Present | 0.04 | 0.19 |
|  | Absent | 0.96 | 0.81 |
| Peripheral Ductal Dilation | Present | 0.04 | 0.02 |
|  | Absent | 0.96 | 0.98 |
| Skin Edema and Thickening | Present | 0.10 | 0.11 |
|  | Absent | 0.91 | 0.90 |
| Axillary Lymph Node Enlargement | Present | 0.91 | 0.93 |
|  | Absent | 0.09 | 0.07 |
| Disappearance of Lymph Node Hilum | Present | 0.40 | 0.50 |
|  | Absent | 0.60 | 0.50 |
| Fat Layer Invasion | Present | 0.84 | 0.70 |
|  | Absent | 0.16 | 0.30 |
| Muscle Layer Invasion | Present | 0.32 | 0.46 |
|  | Absent | 0.68 | 0.54 |
| Long-to-short Axis Ratio > 1 | Present | 0.04 | 0.03 |
|  | Absent | 0.96 | 0.97 |
| Subclavicular Lymph Node Enlargement | Present | 0.31 | 0.32 |
|  | Absent | 0.69 | 0.68 |
| Supraclavicular Lymph Node Enlargement | Present | 0.19 | 0.21 |
|  | Absent | 0.81 | 0.79 |

**Table S7** Predictive Factors for Pathological Non-response Post-chemotherapy in Sensitivity Analysis.

| **Variable** | **OR** | **CI_lower** | **CI_upper** | **p_value** |
| --- | --- | --- | --- | --- |
| Age | 1.01 | 0.95 | 1.08 | 0.68 |
| BMI | 0.91 | 0.79 | 1.05 | 0.21 |
| Menopausal status | 0.53 | 0.16 | 1.73 | 0.29 |
| Family history | 0.51 | 0.08 | 3.12 | 0.47 |
| Reproductive history | 2.34 | 0.39 | 14.03 | 0.35 |
| Chemotherapy regimen | 1.69 | 0.90 | 3.19 | 0.11 |
| ER | 0.93 | 0.25 | 3.42 | 0.91 |
| PR | 0.91 | 0.28 | 2.94 | 0.87 |
| HER2 | 0.95 | 0.38 | 2.38 | 0.92 |
| Ki-67 positivity rate | 1.05 | 0.48 | 2.33 | 0.90 |
| Ultrasound image feature pattern | 2.66 | 1.09 | 6.49 | 0.03 |

**Table S8** Odds Ratios (OR) and 95% Confidence Intervals (CI) for Ultrasound Image Feature Pattern from 10-Fold Cross-Validation in Sensitivity Analysis

| **Fold** | **OR** | **CI_lower** | **CI_upper** | **p-value** |
| --- | --- | --- | --- | --- |
| 1 | 4.01 | 1.35 | 11.94 | 0.01 |
| 2 | 2.83 | 1.15 | 6.94 | 0.02 |
| 3 | 2.59 | 1.05 | 6.36 | 0.04 |
| 4 | 2.32 | 0.88 | 6.13 | 0.09 |
| 5 | 2.64 | 1.05 | 6.61 | 0.04 |
| 6 | 2.38 | 0.91 | 6.21 | 0.08 |
| 7 | 3.10 | 1.21 | 7.93 | 0.02 |
| 8 | 2.68 | 1.09 | 6.60 | 0.03 |
| 9 | 2.75 | 1.12 | 6.74 | 0.03 |
| 10 | 1.83 | 0.71 | 4.71 | 0.21 |

**Table S9** Odds Ratios (OR) and 95% Confidence Intervals (CI) in the Firth Logistic Regression Model (10,000 Bootstrap Resamples) in Sensitivity Analysis

| **Variable** | **OR (95% CI) (mean)** | **% of regression models when variable was significant** | **Average Wald Statistic** |
| --- | --- | --- | --- |
| Age | 1.01 (0.95–1.08) | 0.00% | 0.00 |
| BMI | 0.91 (0.78–1.05) | 0.00% | 0.01 |
| Menopausal status | 0.54 (0.16–1.80) | 0.00% | 0.43 |
| Family history | 0.52 (0.08–3.32) | 0.00% | 0.60 |
| Childbirth history | 2.53 (0.40–16.02) | 0.00% | 0.87 |
| Chemotherapy regimen | 1.73 (0.90–3.32) | 14.00% | 0.31 |
| ER | 0.94 (0.25–3.56) | 0.00% | 0.05 |
| PR | 0.93 (0.28–3.06) | 0.00% | 0.04 |
| HER2 | 0.96 (0.38–2.42) | 0.00% | 0.02 |
| Ki-67 positivity rate | 1.07 (0.48–2.40) | 0.00% | 0.02 |
| Ultrasound Image Feature Pattern | 2.75 (1.11–6.82) | 98.00% | 1.03 |

Abbreviations: BMI, body mass index

**Table S10** Impact of Chemotherapy Regimens on Risk Differences in NAC Response Prediction by Ultrasound Image Feature Patterns in Sensitivity Analysis

| Characteristic Group | Age | BMI | Family history | Childbirth history | Chemotherapy Regimen | Ultrasound image feature pattern risk difference | 95% (CI) |
| --- | --- | --- | --- | --- | --- | --- | --- |
| High Risk | ≥50 years | ≥24 kg/m^2^ | Present | Absent | Taxane | 14.85% | 7.12%, 25.51% |
| High Risk | ≥50 years | ≥24 kg/m^2^ | Present | Absent | Anthracycline | 22.83% | 15.27%, 29.52% |
| High Risk | ≥50 years | ≥24 kg/m^2^ | Present | Absent | Combined | 23.34% | 16.20%, 29.81% |
| Non-high Risk | <50 years | <24 kg/m^2^ | Absent | Present | Taxane | 1.07% | 0.75%, 1.45% |
| Non-high Risk | <50 years | <24 kg/m^2^ | Absent | Present | Anthracycline | 3.26% | 2.41%, 4.28% |
| Non-high Risk | <50 years | <24 kg/m^2^ | Absent | Present | Combined | 3.85% | 2.65%, 5.42% |

Abbreviations: BMI, body mass index


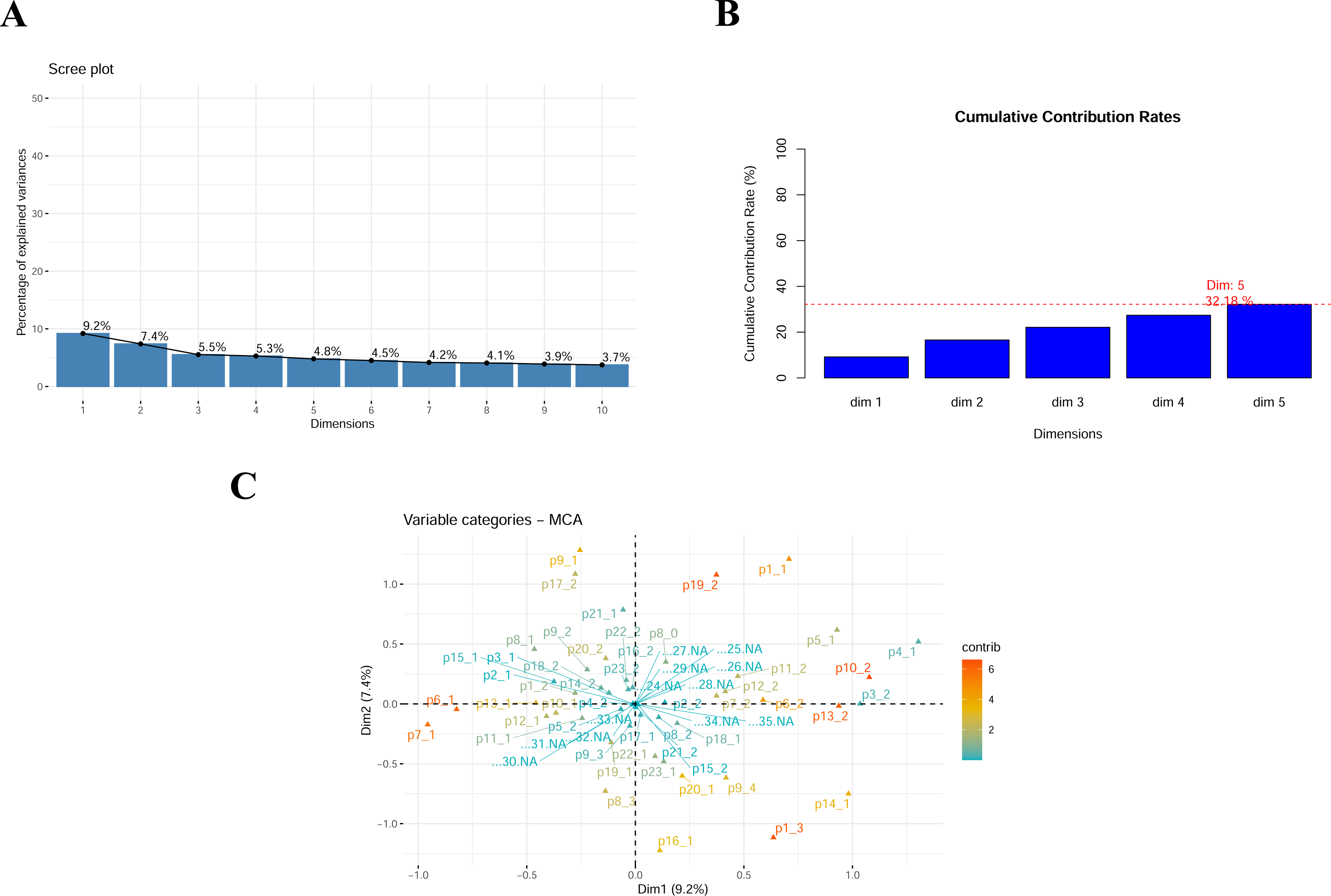


**Fig.S1** MCA Analysis. A. Eigenvalues of the Main Dimensions; B. Cumulative Contribution Rate of Eigenvalues. C Variable Contribution Plot


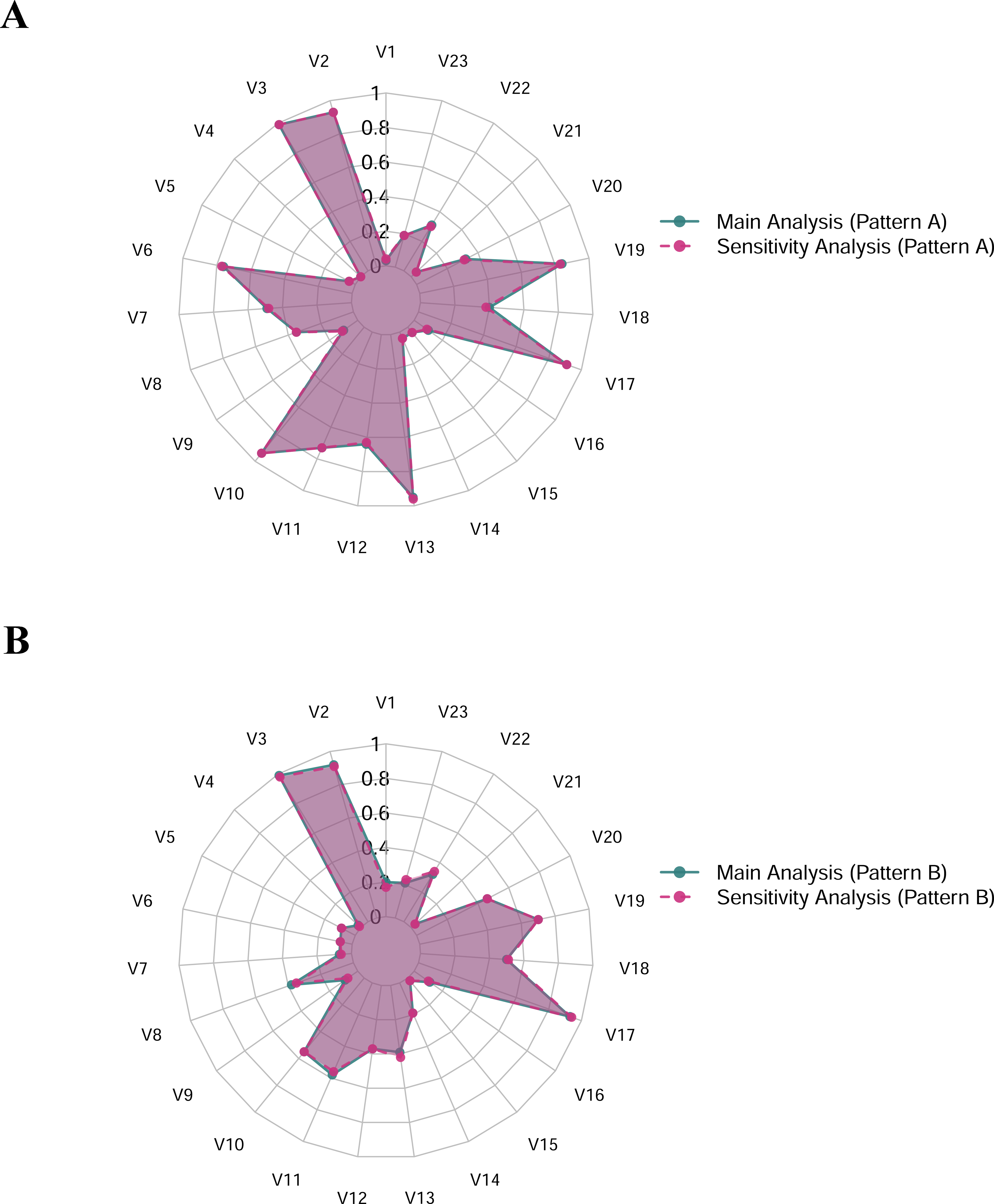


**Fig.S2** Comparison of Ultrasound Imaging Feature Proportions between Main and Sensitivity Analyses for Latent Class Patterns (Note: Each variable takes the first option). A. Pattern A; B. Pattern B.

V1, Tumor Maximum Diameter; V2, Tumor Type; V3, Echo Features; V4, Tumor Shape; V5, Tumor Boundary; V6, Lateral Shadowing; V7, Posterior Echo; V8, Calcification Distribution; V9, Adler Blood Flow Classification; V10, Marginal Angulation; V11, Marginal Spiculation or Crab Claw-like Changes; V12, Peripheral Echogenic Halo; V13, Micro-lobulated Changes; V14, Internal Cystic Area; V15, Peripheral Ductal Dilation; V16, Skin Edema and Thickening; V17, Axillary Lymph Node Enlargement; V18, Disappearance of Lymph Node Hilum; V19, Fat Layer Invasion; V20, Muscle Layer Invasion; V21, Long-to-short Axis Ratio > 1; V22, Subclavicular Lymph Node Enlargement; V23, Supraclavicular Lymph Node Enlargement.
